# Interventions to address unequal gender and power relations and improve self-efficacy and empowerment for sexual and reproductive health decision-making for women living with HIV: A systematic review

**DOI:** 10.1371/journal.pone.0180699

**Published:** 2017-08-24

**Authors:** Jennifer L. Robinson, Manjulaa Narasimhan, Avni Amin, Sophie Morse, Laura K. Beres, Ping Teresa Yeh, Caitlin Elizabeth Kennedy

**Affiliations:** 1 Department of International Health, Johns Hopkins Bloomberg School of Public Health, Baltimore, Maryland, United States of America; 2 Department of Reproductive Health and Research, World Health Organization, Geneva, Switzerland; 3 Department of Health Policy and Management, Johns Hopkins Bloomberg School of Public Health, Baltimore, Maryland, United States of America; University of Toronto, CANADA

## Abstract

**Background:**

Many women living with HIV experience gendered power inequalities, particularly in their intimate relationships, that prevent them from achieving optimal sexual and reproductive health (SRH) and exercising their rights. We assessed the effectiveness of interventions to improve self-efficacy and empowerment of women living with HIV to make SRH decisions through a systematic review.

**Methods and findings:**

We included peer-reviewed articles indexed in PubMed, PsycINFO, CINAHL, Embase, and Scopus published through January 3, 2017, presenting multi-arm or pre-post intervention evaluations measuring one of the following outcomes: (1) self-efficacy, empowerment, or measures of SRH decision-making ability, (2) SRH behaviors (e.g., condom use, contraceptive use), or (3) SRH outcomes (e.g., sexually transmitted infections [STIs]). Twenty-one studies evaluating 11 intervention approaches met the inclusion criteria. All were conducted in the United States or sub-Saharan Africa. Two high-quality randomized controlled trials (RCTs) showed significant decreases in incident gonorrhea and chlamydia. Sixteen studies measuring condom use generally found moderate increases associated with the intervention, including in higher-quality RCTs. Findings on contraceptive use, condom self-efficacy, and other empowerment measures (e.g., sexual communication, equitable relationship power) were mixed. Studies were limited by small sample sizes, high loss to follow-up, and high reported baseline condom use.

**Conclusions:**

While more research is needed, the limited existing evidence suggests that these interventions may help support the SRH and rights of women living with HIV. This review particularly highlights the importance of these interventions for preventing STIs, which present a significant health burden for women living with HIV that is rarely addressed holistically. Empowerment-based interventions should be considered as part of a comprehensive package of STI and other SRH services for women living with HIV.

## Introduction

An increasing body of evidence demonstrates the ways unequal levels of power between men and women in intimate relationships prevent women, including women living with HIV, from making decisions regarding their sexual and reproductive health (SRH) [1–5]. Gender refers to the set of roles, behaviors, and norms that are designated as appropriate for women and men by society [6]. Gender can be the cause, consequence, and/or mechanism of unequal or hierarchical power relations—that is, how power and control are distributed (unequally or hierarchically) in intimate relationships, within the household, in the community, and in wider societal institutions including all the way to the highest levels of political decision-making [6]. In this paper, we focus primarily on the distribution of power in intimate relationships between women and men and within the household. Frequently, unequal control over and access to economic resources, unequal relationship power, and limited ability to make sexual decisions (including whether, when, how often, and with whom to have sex; and negotiating condom use, contraceptive or other protective practices) make women vulnerable to SRH risks [7,8]. Gender inequalities and power imbalances restrict the ability of many women living with HIV to meet their SRH needs and exercise their rights [9].

One approach to address gender inequalities is implementing interventions that seek to empower women living with HIV. Empowerment has been defined as “the process of enhancing the capacity of individuals or groups to make choices and to transform those choices into desired actions and outcomes” [10]. Such interventions are designed to increase women’s self-efficacy, autonomy, or agency, and, hence, improve their sexual and reproductive decision-making and related health outcomes. However, although some interventions have been evaluated on an individual basis, the effectiveness of such interventions as a whole has not been systematically assessed through meta-analyses or systematic reviews.

We conducted a systematic review to examine the effectiveness of interventions that aim to address unequal gender power relations, empower women living with HIV, and increase their self-efficacy to make SRH decisions.

## Methods

This systematic review was conducted to inform World Health Organization guidelines on the sexual and reproductive health and rights of women living with HIV, following PRISMA reporting guidelines [11]; the review protocol is available upon request [12].

### Eligibility criteria

Studies were eligible for inclusion if they met the following criteria:

1. Examined one or more interventions designed to address unequal gender power relations, increase self-efficacy, and/or increase empowerment around safer sex and reproductive decision-making for women living with HIV,
2. Compared women living with HIV who received the intervention to those who did not through a pre/post or multi-arm design,
3. Measured at least one of the following outcomes: (a) Self-efficacy, empowerment, or other measure of ability to make own decisions around condom use, pregnancy termination, birth spacing, childbearing, and other aspects of SRH, (b) SRH behaviors (such as condom use, contraceptive use, disclosure of HIV serostatus to partner) or (c) SRH outcomes (such as STIs, pregnancy).
4. Published in a peer-reviewed journal prior to the search date.

We included studies among all populations of women living with HIV, including adolescents (10–19 years), young people (20–24 years), adults (25+ years), and women of any age who were members of key populations (including female sex workers, women who use drugs, women in prisons or other closed settings, and transgender women) [13]. Given our focus on SRH decision-making, we excluded studies with children under ten years of age. If a study evaluated an intervention for both men and women, or for both women living with HIV and HIV-negative women, it was included only if outcome data were disaggregated for women living with HIV. We did not include self-efficacy for coping with HIV status; self-efficacy for adherence to medications; or general measures of self-efficacy, self-esteem, agency, or wellness not directly linked with SRH behaviors and outcomes. Articles from all countries and written in all languages were eligible for inclusion.

### Data sources

The following electronic databases were searched for articles through January 3, 2017: PubMed, CINAHL, Embase, PsycINFO, and Scopus. We developed search terms for HIV, women, study design, and SRH to identify articles in PubMed (S1 Appendix), then adapted the search for other databases. Secondary reference searching was conducted on all included articles.

### Data analysis

Initial screening of titles and abstracts was conducted by JR and SM. Potentially relevant citations were then independently screened in duplicate by JR and SM and resolved through discussion with CK. Full-text articles were reviewed for final eligibility decisions.

JR and SM independently extracted data in duplicate using standardized forms. Differences in data extraction were resolved through discussion and consensus. The following information was gathered from each included study: objectives, location, population characteristics, intervention description, study design, sample size, follow-up periods, loss-to-follow-up, analytic approach, outcome measures, comparison groups, effect sizes, confidence intervals, significance levels, conclusions, and limitations. JR and CK assessed study rigor using the Evidence Project’s tool for evaluating multiple study designs in HIV behavioral intervention research [14], including assessment of comparison groups, random assignment and selection, follow-up rate, equivalency of comparison groups, and control for potential confounders.

Data were descriptively analyzed by coding categories and SRH outcomes. We did not meta-analyze due to differences in intervention design and outcome measurement across studies. However, we grouped similar measures (e.g., condom use self-efficacy) across studies and summarized findings by outcome.

## Results

**Fig 1:**
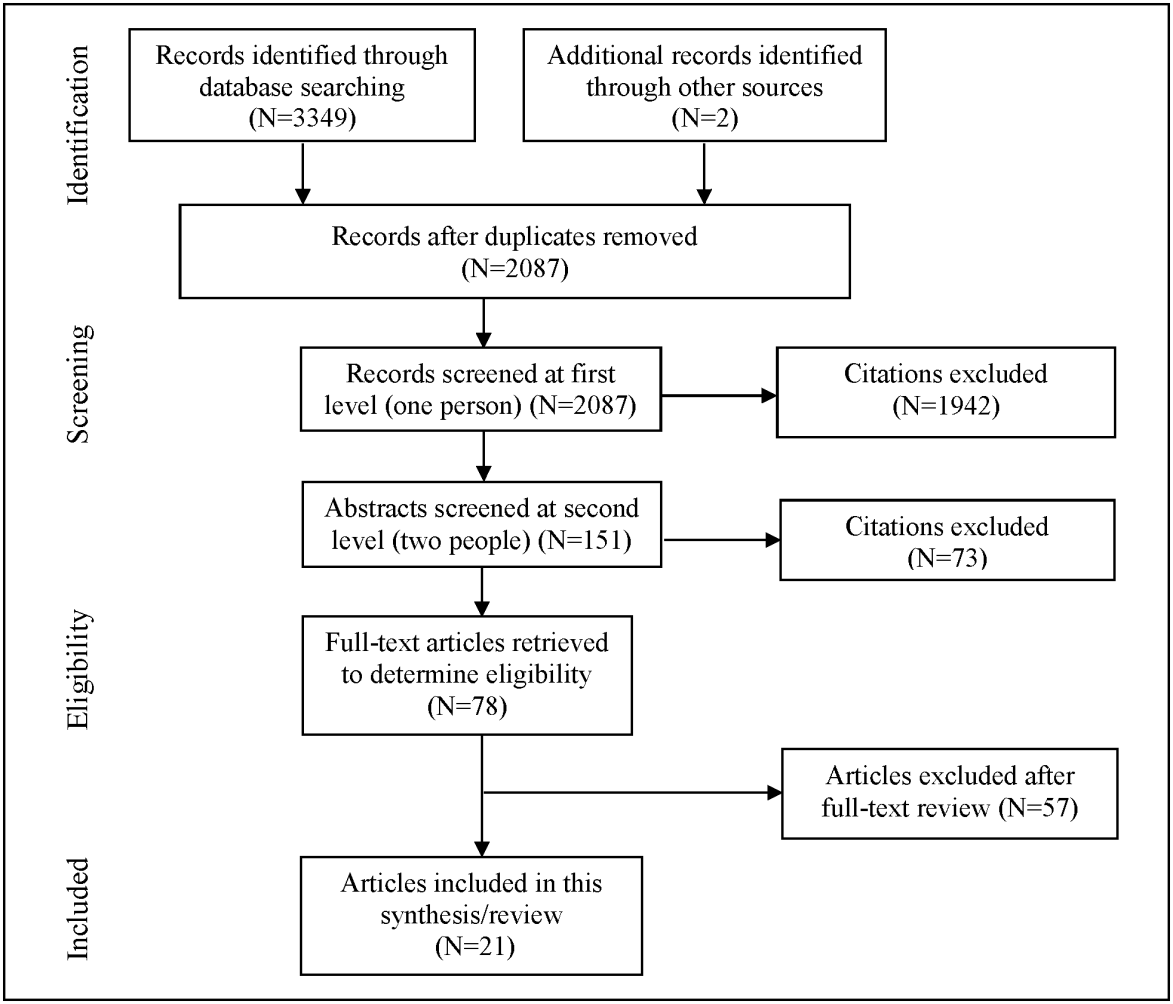
Dispositions of citations through the search and screening process.

Database searches produced a total of 3,351 hits; 2,087 citations remained after removing duplicates (Fig 1). After initial screening, 151 citations were reviewed by two authors in duplicate, of which 73 were excluded for not meeting the inclusion criteria (e.g., qualitative studies, studies without relevant outcomes, or studies without findings for women living with HIV). Seventy-eight articles were pulled for full-text review, and 57 were excluded. Ultimately, 21 studies were included in the review covering 11 specific intervention approaches (Table 1).

**Table 1:** Descriptions of included studies.

*Author Year Location*	*Sexual and Reproductive Health and Rights Empowerment Intervention*	*Study Design*	*Intervention Provider*	*Theoretical Framework*	Study Outcomes*
**SISTA Adaptations (WiLLOW, PURSE)**					
Wingood, et al., 2004					
Atlanta, GA; Birmingham, AL, USA	**WiLLOW**: ‘Women involved in life learning from other women’ 4, 4-hour interactive group sessions implemented over consecutive weeks with 8–10 group participants. Topics Covered: Gender pride; supportive social network use and maintenance; HIV transmission risk behaviors, communication and safe sex negotiation, condom use, managing abusive relationships *Primary Objective*: *Reduce unprotected vaginal sex*	RCT, individual N = 366 Follow-Up Time: 12 months	Trained female health educator, co-facilitated by HIV-positive female peer educator	Social cognitive theory; Theory of gender and power; designed for women living with HIV	Unprotected vaginal intercourse Proportion never used condoms Incident STDs Condom Self-Efficacy
Saleh-Onoya et al., 2009					
Western Cape, South Africa	**WiLLOW Adaptation** 4, 4-hour group sessions implemented over consecutive weeks with 8–10 group participants Topics Covered: sexual risk reduction and coping training (e.g., ethnic and gender pride, self-esteem, support networks, communication, HIV risk behaviors, etc.) *Primary Objective*: *Enhance coping skills and consistent condom use*	RCT, individual N = 120 Follow-Up Time: 3 months	Black, isiXhosa speaking, female health educator and a black isiX- hosa speaking HIV-positive woman co-facilitator	Social cognitive theory; Theory of gender and power; designed for women living with HIV	Self-efficacy for negotiating condom use Self-efficacy for correct condom use Control in relationships Condom use at last sex STI Incidence
Klein et al., 2013					
Southern USA	**Multimedia WiLLOW** 2, 1-hour interactive computer session separated into 2–8 minute activity modules Topics covered: pride, values, goals, using social support, stress management, risk reduction, condom management, building healthy relationships, HIV re-infection, STIs, partner communication, disclosure, condom self-efficacy, computer use instructions *Primary Objective*: *Increase protective sexual behaviors and psychosocial mediators associated with HIV risk reduction*	RCT, individual N = 175 Follow-Up Time: 3 months	Interactive computer modules with female African American narrator	Social cognitive theory; Theory of gender power, built from each piece of WiLLOW meetings	Condom Use Partner sexual communication Communication self-efficacy
Sarnquist et al., 2014					
Chitungwiza, Zimbabwe	**PURSE**: ‘Peers Undertaking Reproductive and Sexual Health Education’ 3, 90-minute group sessions Topics covered: sexual negotiation skills and empowerment, information about HIV, PMTCT, and FP, and communication skills related to sex and FP. *Primary Objective*: *Achieve desired family size and spacing; maximize maternal and child health*	Non-randomized trial N = 98 Follow-Up Time: 3 months postpartum	Nurses with enhanced FP training	Social learning theory, Theory of gender and power	Relationship power Control over condom use Long-acting reversible contraception (LARC) use
**SWP ‘SMART/EST Women’s Project’; NOW/NOW2; The Partner Project**					
Jones et al., 2001					
Miami, FL; Newark, NJ; New York, NY, USA	**NOW**: ‘New Opportunities for Women’ 3, 120-minute sessions over 3 months Topics Covered: HIV/STD transmission, hierarchical counseling, skill training, reactions to barriers, cognitive reframing, and sexual negotiation *Primary Objective*: *Increase sexual barrier use*	Non-randomized trial with matched controls N = 178 Follow-Up Time: 9 months	Psychologist	Hierarchical approach	Use of N-9 spermicides
Jones et al., 2005					
Lusaka, Zambia	**The Partner Project** (NOW Adaptation) 4 group intervention sessions; male partners attended 1 or 4 separate sessions Topics covered: HIV/sexually transmitted disease prevention and transmission, reproductive choice and mother to child transmission, communication, conflict resolution, sexual negotiation *Primary Objective*: *Reduce sexual risk behavior*	RCT, individual N = 332 (180 women living with HIV) Follow-Up Time: 12 months	Trained gender-congruent counselors	Theory of reasoned action/planned behavior	Protected sex
Jones et al., 2006					
Lusaka, Zambia	**NOW2** (NOW Adaptation) 2-hour group sessions limited to 10 women Topics covered: (1) HIV/STDs, safer sex, barrier use, reproductive choice, HIV re-infection, transmission and infection with other STDs and hierarchical methods of sexual barrier use (2) Vaginal lubricants, gels and suppositories *Primary Objective*: *Increase sexual barrier use*	RCT, individual N = 240 Follow-Up Time: 12 months	Registered and licensed practical nurses and healthcare staff trained in intervention administration	Theory of reasoned action/planned behavior	Sexual barrier use Male condom use Female condom use
Jones et al., 2007					
Miami, FL, USA	**NOW** ‘New Opportunities for Women’ 3, 120-minute sessions over 3 months limited to 10 participants Topics covered: sexual barrier products, sexual risk reduction strategies, sexual negotiation *Primary Objective*: *Increase sexual barrier use*	Randomized trial without control (randomized to individual or group sessions) N = 187 Follow-Up Time: 12 months	Facilitators were gender matched RNs, LPNs and health care staff trained in the administration of each condition	Theory of reasoned action/planned behavior	Risk behavior
Weiss et al., 2011					
Miami, FL; Newark, NJ; New York, NY, USA	**SWP I and II ‘SMART/EST Women’s Program’ + Group Healthy Living Component** 10 weekly 2-hour group cognitive—behavioral stress management/expressive—supportive therapy framework (CBSM+) 6 additional 2-hour group healthy living sessions Topics covered: medication adherence, nutrition, safer sex, substance abuse reduction, and physical activity. *Primary Objective*: *Optimize health status of poor women of color living with HIV*	RCT, individual N = 933 Follow-Up Time: 24 months	Psychologist	Cognitive behavioral stress management (CBSM) plus expressive supportive therapy framework (CBSM+)	Unprotected sex Vaginal sexual barriers
Jones et al., 2013					
Miami, FL; Newark, NJ; New York, NY, USA	**SWP ‘SMART/EST Women’s Program’ Community Health Center Adaptation** 10 weekly 2-hour group cognitive—behavioral stress management/expressive—supportive therapy framework (CBSM+) 6 additional 2-hour group healthy living sessions Topics covered: medication adherence, nutrition, physical activity, sexual risk behavior, and alcohol and drug use *Primary Objective*: *Optimize health status of women living with HIV in a community health setting*	Non-randomized trial N = 428 Follow-Up Time: 12 months	Health-care providers, counselors, social workers, and health educators	Cognitive behavioral stress management (CBSM) plus expressive supportive therapy framework (CBSM+), Glasgow's RE-AIM model	Number of sexual partners
**M2M ‘Mothers 2 Mothers’; Mamekhaya**					
Futterman et al., 2010					
Peri-urban Cape Town, South Africa	**Mamekhaya, based on M2M ‘Mothers 2 Mothers’** 8 session, small groups of pregnant women Topics Covered: Healthy Living- staying in care, dealing with symptoms, HIV, ARVs, family planning, condoms; Feeling Happy & Strong- disclosure, stigma, support, hope, negative emotions, domestic violence, substance abuse; Partnering & Preventing Transmission: infant feeding, partner testing, safer sex; Parenting: feeding, immunizations, infant testing, custody, attachment; in all sessions: music, meditation, active learning *Primary Objective*: *PMTCT and maternal well-being*	Non-randomized trial, group N = 160 Follow-Up Time: 6 months post-delivery	M2M mentor mothers (women living with HIV) trained in CBI	Cognitive behavioral interventions, empowerment and support model	Partner testing Abstinence/condom use
Richter et al., 2014					
KwaZulu-Natal, South Africa	**Mothers2Mothers Adaptation** 8 individual mentor sessions: 4 antenatal, 4 postnatal Topics covered: destigmatizing HIV, PMTCT tasks, exclusive feeding, abstaining from traditional medicines, healthy daily routines, obtaining a child grant, maintaining strong social network, couples’ HIV testing, disclosure, condom use *Primary Objective*: *Maternal and infant well-being*	RCT, group N = 1,200 Follow-Up Time: 1.5 months post-birth	Peer mentors	Empowerment and support model	Asking partner to test for HIV
**KHARMA ‘Keeping Healthy and Active with Risk Reduction and Medication Adherence’**					
Holstad et al., 2011					
A large southeastern metropolitan city, USA	**KHARMA** ‘Keeping Healthy and Active with Risk Reduction and Medication Adherence’ 8 group sessions Topics covered: ART adherence, risk behavior, HIV status disclosure *Primary Objective*: *Promotion of adherence to antiretroviral medications and risk reduction behaviors*	RCT, individual N = 203 Follow-Up Time: 9 months	Trained nurses	Motivational interviewing theory	Abstinence Use of Protection
Holstad et al., 2012					
Lagos, Nigeria	**KHARMA Adaptation** 8 group sessions Topics covered: ART adherence, self-efficacy for condom skills and knowledge, condom negotiation, HIV status disclosure *Primary Objective*: *Promotion of adherence to antiretroviral medications and risk reduction behaviors*	RCT, individual N = 60 Follow-Up Time: 6 months	Trained nurses	Motivational interviewing theory; Social cognitive theory	Number of sexual partners Use of condoms/protection Drug/alcohol use prior to sex
**HR ‘Healthy Relationships’**					
Marhefka et al., 2014					
Florida, USA	**HR-VG** ‘Healthy Relationships—Videoconferencing Groups’ (HR Adaptation) 6, 2-hour videoconference sessions Topics covered: HIV status, disclosure decision-making and safer sexual behaviors *Primary Objective*: *Reducing sexual risk behavior*	RCT, individual N = 71 Follow-Up Time: 6 months	2 women living with HIV (1 social worker, 1 community member)	Social cognitive theory	Unprotected sex
**Project ROADMAP ‘Reeducating Older Adults in Maintaining AIDS Prevention**’					
Echenique et al., 2013					
Miami, FL USA	**ROADMAP** ‘Reeducating Older Adults in Maintaining AIDS Prevention’ 4 weekly psycho-educational group sessions for older women, 2-hours each Topics covered: HIV, harm reduction, effects of HIV on sexual behaviors, assertive communication with partners, condom negotiation, de-escalating negative partner reactions, review of lessons learned, self-reward for maintaining safer behavior *Primary Objective*: *Reduce high risk sexual behavior*	RCT, individual N = 300 Follow-Up Time: 6 months	Peer educators	Information-motivation-behavioral skills (IMB model) of AIDS risk behavior change; principles of self-efficacy theory	Condom use
**WDIP ‘Women and Infants Demonstration Project’**					
Fogarty et al., 2001					
Baltimore, MD, USA	**WDIP** ‘Women and Infants Demonstration Project’ Unlimited individual sessions over 6 month period Topics covered: condom and contraceptive use, condom negotiation *Primary Objective*: *condom and contraceptive use*	RCT, individual N = 322 Follow-Up Time: 18 months	Trained peer mentors	Stages of change theory	Condom use self-efficacy Condom use Contraceptive use
**Protect and Respect**					
Teti et al., 2010					
Philadelphia, PA, USA	**Protect and Respect** 5 consecutive, weekly, 1.5 hour group intervention sessions and peer-led support groups Topics covered: sexual risk reduction education and skill-building; women’s challenges and opportunities; HIV/AIDS and STI facts; male and female condom use and condom negotiation; triggers to unsafe sex; HIV status disclosure; problem solving; healthy relationships; social support; and goal setting. *Primary Objective*: *increase HIV status disclosure and condom use*	RCT, individual N = 184 Follow-Up Time: 18 months	Health care professionals, health educators, and peer educators	Transtheoretical model of the stages of change; Modified AIDS risk reduction model; Theory of gender and power; formative research	Condom use
**WHC ‘Women’s Health CoOp’**					
Wechsberg et al., 2010					
Pretoria, South Africa	**WHC-Pretoria** ‘Women’s Health CoOp’ (WHC Adaptation) 2 individual 1-hour sessions held within a 2-week period Topics covered: substance abuse, HIV/STIs, HIV risk, behavioral skills training with condoms, violence prevention, sexual negotiation and communication *Primary Objective*: *reduce sexual risk*, *substance use*, *and victimization among at-risk and underserved women*	RCT, individual N = 214 Follow-Up Time: 6 months	Trained interventionist	Gender and empowerment theories	Condom use
**ESHI ‘Enhanced Sexual Health Intervention’**					
Wyatt et al., 2004					
Los Angeles, CA, USA	**ESHI** ‘Enhanced Sexual Health Intervention’ 11 weekly 2.5-hour psycho-educational sessions Topics covered: HIV risk behaviors, interpersonal and health behaviors, and psychological symptoms *Primary Objective*: *reduce sexual risks and increase HIV medication adherence for HIV-positive women with child sexual abuse (CSA) histories*	RCT, individual N = 147 Follow-Up Time: 6 months	Trained group facilitator and peer mentor living with HIV with a history of CSA	Cognitive-behavioral approaches to risk reduction and cultural- and gender-specific concepts	Condom use
**EVOLUTION**					
Brothers et al., 2016					
Baltimore, MD; Chicago, IL; Tampa, FL, USA	**EVOLUTION**: Young Women Taking Charge and Growing Stronger 9 (7 group, 2 individual) weekly 2–3 hour sessions with 6–8 women per group Topics covered: HIV risk reduction education and sexual negotiation skills, forgiveness, emotional regulation, communication, relationships *Primary Objective*: *Decrease sexual risk; empower young women living with HIV through knowledge and skills*	RCT, individual N = 43 Follow-Up Time: 3 months	Trained group facilitator	Theory of gender and power	Sexual activity and sexual risk questionnaire Self-efficacy for limiting HIV risk behavior Self-efficacy for sexual discussion Condom use self-efficacy Sexual beliefs

*Only outcomes relevant to self-efficacy and empowerment around sexual and reproductive health are included.

### Study descriptions

#### Location

Thirteen studies were located in the United States (US) [15–27], while eight were adapted from US-based interventions to an African context, including four in South Africa [28–31], two in Zambia [32,33], one in Zimbabwe [34], and one in Nigeria [35]. The US-based studies were largely implemented in urban areas. Two studies did not specify the exact study location, but were located in “a southern state with a high HIV prevalence” and “a large southeastern metropolitan city” respectively [16, 20].

#### Population characteristics

All studies included women living with HIV, per our inclusion criteria. Several studies focused on women from vulnerable or key populations, such as women with high rates of alcohol and other drug use [19,31], female sex workers [31], pregnant women [28,29,34], older adults [15], young women [27], and women with histories of child sexual abuse [26]. The US-based studies included primarily African-American and Hispanic women [15–26]. Across studies, ages ranged from 16–70 years old.

#### Study design

Tables 1 and 2 present information on study design and quality assessment. Sixteen studies were randomized controlled trials (RCTs) with randomization at either the individual or group (facility/community) level [15–17,21–27,29–33,35], while five studies employed other study designs, including non-randomized trials and a randomized trial with no control (participants randomized to group or individual intervention) [18–20,28,34]. Sample sizes at baseline ranged from 43 to 1,200; several of the smaller studies were described as feasibility or pilot studies. Follow-up time ranged from 3–24 months. Ten studies had follow-up rates of 75% or more.

**Table 2:** Quality assessment of included studies.

*Author*, *Year*	*Cohort*	*Control or comparison group*	*Pre/post intervention data*	*Random assignment of participants to the intervention*	*Random selection of participants for assessment*	*Follow-up rate of 75% or more*	*Comparison groups equivalent socio-demographics*	*Comparison groups equivalent at baseline on outcome measure*	*Control for potential confounders*
**SISTA Adaptations (WiLLOW, PURSE)**									
Wingood et al., 2004	Yes	Yes	Yes	Yes	No	Yes	Yes	Yes	Yes
Saleh-Onoya et al., 2009	Yes	Yes	Yes	Yes	No	Yes	Yes	No	Yes
Klein et al., 2013	Yes	Yes	Yes	Yes	No	Yes	Yes	Yes	Yes
Sarnquist et al., 2014	Yes	Yes	Yes	No	No	Yes	Yes	Yes	Unclear
**SWP ‘SMART/EST Women’s Project’; NOW/NOW2; The Partner Project**									
Jones et al., 2001	Yes	Yes	Yes	No	No	No	Yes	Yes	No
Jones et al., 2005	Yes	Yes	Yes	Yes	No	Unclear	Unclear	Unclear	No
Jones et al., 2006	Yes	Yes	Yes	Yes	No	No	Yes	Yes	No
Jones et al., 2007	Yes	Yes	Yes	Yes	No	Unclear	Unclear	Unclear	No
Weiss et al., 2011	Yes	Yes	Yes	Yes	No	Unclear	Unclear	Unclear	No
Jones et al., 2013	Yes	Yes	Yes	Unclear	No	Yes	No	No	No
**M2M ‘Mothers 2 Mothers’; Mamekhaya**									
Futterman et al., 2010	Yes	Yes	No	No	No	No	No	Unclear	Yes
Richter et al., 2014	Yes	Yes	Yes	Yes	No	No	Yes	Yes	Unclear
**KHARMA ‘Keeping Healthy and Active with Risk Reduction and Medication Adherence’**									
Holstad et al., 2011	Yes	Yes	Yes	Yes	No	Yes	No	Unclear	Yes
Holstad et al., 2012	Yes	Yes	No	Yes	No	Yes	No	Unclear	Unclear
**HR ‘Healthy Relationships’**									
Marhefka et al., 2014	Yes	Yes	Yes	Yes	No	Yes	Yes	Yes	Yes
**Project ROADMAP ‘Reeducating Older Adults in Maintaining AIDS Prevention**’									
Echenique et al., 2013	Yes	Yes	Yes	Yes	No	No	Yes	Yes	No
**WDIP ‘Women and Infants Demonstration Project’**									
Fogarty et al., 2001	Yes	Yes	Yes	Yes	No	No	Unclear	Unclear	Unclear
**Protect and Respect**									
Teti et al., 2010	Yes	Yes	Yes	Yes	No	No	Yes	Yes	Yes
**WHC ‘Women’s Health CoOp’**									
Wechsberg et al., 2010	Yes	Yes	Yes	Yes	No	No	Unclear	Unclear	Yes
**ESHI ‘Enhanced Sexual Health Intervention’**									
Wyatt et al., 2004	Yes	Yes	Yes	Yes	No	Yes	Yes	Yes	Yes
**EVOLUTION**									
Brothers et al., 2016	Yes	Yes	Yes	Yes	No	Yes	Yes	Yes	Yes

#### Theoretical bases

All programs had an underlying theoretical basis. Theories used included social learning theory/social cognitive theory [15,21,22,25,30,34,35], the theory of gender and power [21,23,25,27,30,31,34], the empowerment and support model [28,29], the theory of reasoned action and theory of planned behavior [19,32,33], stages of change theory [16,23], and the AIDS risk reduction model [23].

#### Intervention descriptions

The 21 included studies covered 11 specific interventions (Table 1). Some interventions included multiple adaptations to different geographic context. In one instance, an in-person intervention was later adapted for multimedia [21]. Several were considered effective behavioral interventions by the U.S. Centers for Disease Control and Prevention.

Interventions were generally delivered in small group or one-on-one sessions. Several interventions incorporated cognitive-behavioral components, including cognitive-behavioral stress management/expressive-supportive therapy and cognitive-behavioral skill training [18,24,26,28]. Motivational interviewing was also common [15,17,35,36].

### Study outcomes

Table 3 presents study outcomes. Two studies measured STI incidence [25,30]. Eighteen of the 21 studies measured sexual and reproductive health behaviors: 16 measured condom use [15–17,21–28,30–33,35] while two measured contraceptive use [16,34]. Six studies measured self-efficacy and psychosocial outcomes [16,21,25,27,30,34]. No studies measured reproductive health decision-making around pregnancy termination, birth spacing, or childbearing.

**Table 3:** Sexual and reproductive health findings from included studies.

*Author Year*	Study Findings*	
**SISTA Adaptations (WiLLOW, PURSE)**		
Wingood et al., 2004	**Condom use self-efficacy:** % relative change comparing intervention to control: 8.1 (95% CI 1.1, 15.0), p = 0.001 Adjusted mean difference: 1.0 (95% CI 0.2, 1.9) **Number of acts of unprotected vaginal sex, past 30 days:** % relative change comparing intervention to control: -28.0 (95% CI -69.3, -13.4), p = 0.022 Adjusted mean difference: -0.7 (95% CI -1.8, -0.4)	**Proportion never used condoms, past 30 days:** OR = 0.3 (95% CI 0.1, 0.7), p = 0.008 **Incident bacterial STD (chlamydia or gonorrhea):** OR = 0.2 (95% CI 0.1, 0.6), p = 0.006 Incident bacterial *Trichomonas* infection: No differences observed, no data reported
Saleh-Onoya et al., 2009	Condom use self-efficacy: F = 1.65, p = 0.20 Self-efficacy for negotiating condom use: F = 0.47, p = 0.50 Relationship power: F = 0.77, p = 0.38 Condom use at last sex: OR = 0.48 (95% CI 0.09, 2.54), p = 0.39	Incident bacterial vaginosis: OR = 1.23 (95% CI 0.53, 2.85) **Incident trichomonas vaginalis:** OR = 0.06 (95% CI 0.01, 0.46) **Incident gonorrhea:** OR = 0.10 (95% CI 0.02, 0.49) **Incident chlamydia:** OR = 0.21 (95% CI 0.07, 0.59)
Klein et al., 2013	**Sexual communication self-efficacy:** % relative change comparing intervention to control: 9.70 (95% CI 2.08, 21.77), p = 0.004 Adjusted mean difference: 3.40 (95% CI 1.12, 5.65) **Condom-protected vaginal and anal sex acts, past 30 days:** % relative change comparing intervention to control: 45.21 (95% CI 17.67, 71.36), p = 0.002 Adjusted mean difference: 0.33 (95% CI 0.13, 0.52)	**100% condom use:** OR = 9.67 (95% CI 1.25, 74.97), p = 0.30 **Number of unprotected vaginal and anal sex acts, past 30 days:** % relative change comparing intervention to control: -133.67 (95% CI -190.20, -41.71), p = 0.002 Adjusted mean difference: -3.41 (95% CI -5.54, -1.29)
Sarnquist et al., 2014	**Relationship power:** Intervention: 2.5%, Control: 2.1%, p = 0.01 **Control over condom use:** Intervention: 67.2%, Control: 34.4%, p = 0.002 Use of long-acting reversible contraception: Intervention: 87.1%, Control: 81.8%, p = 0.34	**Disclosure of HIV serostatus, woman to partner:** Intervention: 98.4%, Control: 87.5, p = 0.04 **Disclosure of HIV serostatus, partner to woman:** Intervention: 75.8%, Control: 55.2%, p = 0.04
**SWP ‘SMART/EST Women’s Project’; NOW/NOW2; The Partner Project**		
Jones et al., 2001	**Use of N-9 spermicides:** Intervention: 83%, Control: 9%, p<0.05	
Jones et al., 2005	**Protected sex, 6 months after baseline:** X = 4.90, t(1,70) = -.67, p<0.001	**Protected sex, 12 months after baseline:** X = 4.83, t(1,30) = -3.20, p = 0.003
Jones et al., 2006	**Male condom use, 6 months after baseline:** Group vs individual intervention: F = 13.5, p<0.001 Male condom use, 12 months after baseline: Group vs individual intervention: F = 0.24, p = 0.62	**Sexual barrier use, 6 months after baseline:** Group vs individual intervention: F = 4.6, p<0.05 Sexual barrier use, 12 months after baseline: Group vs individual intervention: F = 0.5, p = 0.05
Jones et al., 2007	Sexual risk behavior: Group vs individual intervention: F = 1.31, p = 0.27	
Weiss et al., 2011	**Unprotected sex:** Decreased OR from 0.16 to 0.095, F = 0.04, p = 0.038	
Jones et al., 2013	**Number of sex partners:** OR = 0.6 (95% CI 0.4–0.9)	
**M2M ‘Mothers 2 Mothers’; Mamekhaya**		
Futterman et al., 2010	Abstinent or always uses condom: Coefficient: 0.24, SE: 1.44, p>0.05	
Richter et al., 2014	**Asking partners to test for HIV:** OR = 1.84, p = 0.014	
**KHARMA ‘Keeping Healthy and Active with Risk Reduction and Medication Adherence’**		
Holstad et al., 2011	**Always uses condoms, past 3 months:** Z = 2.10, p = 0.036	
Holstad et al., 2012	**Always uses condoms, past 3 months:** Intervention: 84.6%, Control: 43.8%, p = 0.014	**Condom use at last sexual encounter:** Intervention: 88.9%, Control: 52.6%, p = 0.015
**HR ‘Healthy Relationships’**		
Marhefka et al., 2014	Proportion reporting no unprotected sex, past 3 months: OR = 0.92 (95% CI 0.24, 3.56)	Difference in frequency of unprotected sex, past 3 months: Difference = 6.89 (95% CI 5.43, 8.73)
**Project ROADMAP ‘Reeducating Older Adults in Maintaining AIDS Prevention**’		
Echenique et al., 2013	**Inconsistent condom use with all partners:** Intervention: 20% at baseline; 9.2% at 6-months, p = <0.05 Comparison: 12.2% at baseline; 9.8% at 6-months, p = 0.42 **Inconsistent condom use with HIV-negative/unknown serostatus partners:** Intervention: 12.3% at baseline; 3.1% at 6-months, p<0.10 Comparison: 2.4% at baseline; 4.9% at 6-months, p = 0.51	Inconsistent condom use with HIV-positive partners: Intervention: 7.7% at baseline; 6.2% at 6-months, p>0.99 Comparison: 9.8% at baseline; 9.8% at 6-months, p>0.99
**WDIP ‘Women and Infants Demonstration Project’**		
Fogarty et al., 2001	**Self-efficacy for condom use with main partner:** OR = 2.01, p = 0.01 Progress** in use of condoms with main partner: OR = 2.30, p = 0.02	Progress** in use of contraceptives: OR = 2.07, p = 0.08 Relapse in use of contraceptives: OR = 0.43, p = 0.03
	**Progress in terms of Stages of Change theory: moving up a stage or staying in maintenance	
**Protect and Respect**		
Teti et al., 2010	**Proportion of sex acts where condoms used:** Difference in OR = 270.04 (95% CI: 24.53, 2971.94), p<0.01	
**WHC ‘Women’s Health CoOp’**		
Wechsberg et al., 2010	**Condom use at last sex act:** OR = 7.27 (95% CI 1.64, 32.23), p<0.05	
**ESHI ‘Enhanced Sexual Health Intervention’**		
Wyatt et al., 2004	**Condom use with main partner, past 3 months:** OR = 2.96, p = 0.039	
**EVOLUTION**		
Brothers et al., 2016	Number of male partners, past 3 months: RR = 1.11 (95% CI 0.72, 1.70), p = 0.648 Any unprotected vaginal or anal intercourse, past 3 months: Adjusted OR = 0.26 (95% CI 0.05, 1.51), p = 0.135 Self-efficacy for limiting HIV risk behavior Adjusted mean difference: 0.04 (95% CI -0.14, 0.21), p = 0.667	Self-efficacy for sexual discussion Adjusted mean difference: -0.16 (95% CI -0.36, 0.04), p = 0.110 Condom use self-efficacy Adjusted mean difference: 0.14 (95% CI -0.10, 0.37), p = 0.250 Sexual beliefs Adjusted mean difference: 0.05 (95% CI -0.15, 0.24), p = 0.631

*Bold indicates significant difference between intervention and comparison groups.

Odds ratios represent odds in the intervention group compared to the control group.

#### Sexually transmitted infections

Two studies measured STI incidence: the original WiLLOW intervention in the southern USA and its South African adaptation. Both were high-quality RCTs, although the South African adaptation had a shorter follow-up time (3 vs. 12 months) and smaller sample size (102 vs. 321 participants) [25,30]. Both studies showed significant decreases in STI incidence. The original WiLLOW intervention found a significant reduction in incidence of bacterial STIs (*Chlamydia trachomatis* and gonorrhea) over 12-month follow-up in intervention versus control participants (OR = 0.20, 95% CI = 0.10–0.60). However, there was no significant change in *Trichomonas vaginalis* [25]. In the South African adaptation, the intervention group similarly showed a significant reduction in incidence of *Chlamydia trachomatis* (OR = 0.21, 95% CI = 0.07–0.59) and gonorrhea (OR = 0.10, 95% CI = 0.02–0.49) compared to the control group. The South African adaptation further showed a significant decrease in incidence of *Trichomonas vaginalis* (OR = 0.06, CI = 0.01–0.46), but no difference in incidence of bacterial vaginosis [30].

#### Condom use

Sixteen studies (11 interventions) measured condom use [15–17,21–28,30–33,35]; however, studies used a wide range of measures, precluding meta-analysis. These studies (12 RCTs and four other designs) showed mixed results. Although most studies found significant increases in condom use, others found no change and increases were often moderate, often affected by high background rates of condom use.

Of the three SISTA adaptation RCTs that measured condom use, two showed significant increases [21,25], while the South African adaptation did not [30]. Most other high-quality RCTs also found significant positive impacts on condom use by various measurements [17,24,26,31–33,35]. WDIP found progress (through stages of change) in condom use with main partner [16]. Three studies with high loss to follow-up rates (30–44% retention at follow-up) found mixed results on condom use [15,23,28].

#### Contraceptive use

Two studies measured contraceptive use. WDIP, an RCT, found that intervention participants were more likely to show progress (OR = 2.07, p = 0.08) and significantly less likely to relapse (OR = 0.43, p = 0.03) in contraceptive use compared to the comparison group [16]. PURSE, a non-randomized trial with 98 participants and high rates of follow-up, found that uptake of long-acting reversible contraception increased in both intervention and control groups three months after delivery, but there was no significant difference across groups (I: 87%, C: 81.8%, p = 0.34). The authors suggested this was due to both groups having access to nurses with training in enhanced family planning [34].

#### Self-efficacy and psychosocial measures

Four RCTs (the original WiLLOW, its South African adaptation, WDIP, and EVOLUTION) and one non-randomized trial (PURSE) measured condom use self-efficacy. The original WiLLOW program found that intervention participants had higher condom use self-efficacy over 12 months of follow-up (13.6 vs. 12.6; p = 0.001) [25]. PURSE also found significant increases in self-reported control over condom use (67.2% vs. 34.4%, p = 0.002) [34], and WDIP intervention participants showed higher self-efficacy for condom use with a main partner than control participants (OR = 2.01, p = 0.01) [16]. However, neither the small EVOLUTION pilot study nor the South African WiLLOW adaptation found a significant difference between intervention and control groups in condom use self-efficacy [30].

Other psychosocial outcomes also showed mixed results. The multimedia WiLLOW adaptation reported improvement in sexual communication self-efficacy (mean difference = 3.40, p = 0.004) [21], while EVOLUTION found no significant impacts on self-efficacy for sexual discussion or self-efficacy for limiting HIV risk behavior [27]. PURSE found significant increases in relationship power (2.5 vs. 2.1, p = 0.01) [34], whereas the South African WiLLOW adaptation found no significant results for relationship control or condom negotiation [30]. Finally, PURSE intervention participants were more likely to report disclosing their HIV status to a partner (98.4% vs. 87.5%, p = 0.04) and vice versa (75.8% vs. 55.2%, p = 0.04) [34].

## Discussion

All women living with HIV must be supported in their voluntary choices around sexual relationships and be given information and resources to engage in safe, enjoyable sexual experiences, or to not engage in sex based on their personal preference, with counselling and support tailored to their decision-making, desires and needs. Supporting women living with HIV in all their diversity to achieve their sexual and reproductive health and rights in all epidemic contexts requires overcoming major barriers to service uptake such as social exclusion and marginalization, criminalization, stigma, and gender inequality [37]. Addressing unequal gender and power relations and empowering women living with HIV may be one part of a comprehensive approach to achieve these goals.

This systematic review highlights the potential for increasing condom use and reducing incident STIs through empowerment interventions for women living with HIV. STIs continue to be an important public health issue that can facilitate sexual transmission of HIV and trigger some cancers. As stated in the WHO Global Health Sector Strategy on Sexually Transmitted Infections, 2016–2021, “the burden of morbidity and mortality worldwide resulting from sexually transmitted pathogens compromises quality of life, as well as sexual and reproductive health” [37]. Women living with HIV have high rates of STI co-infection, with a mean STI prevalence of 15.8% (standard deviation: 9.9) across studies in a recent global systematic review [38]. Although STI screening and treatment are a recommended part of the package of care for people living with HIV by the WHO [39,40] and PEPFAR [41], a comprehensive, rights-based approach to addressing STIs and other SRH issues is needed to facilitate STI prevention as well as treatment for women living with HIV.

Findings from our review were more mixed, however, for other outcomes, including contraceptive use, self-efficacy, and psychosocial measures. While these interventions hold promise, further work is needed to determine which components of interventions make them successful, for which populations, and on which outcomes.

Conclusions from this review are limited by the nature of the evidence base. The range of outcomes measured by the included studies was narrow, with the majority measuring condom use. Only a few studies measured other SRH outcomes, or more proximal outcomes such as empowerment and self-efficacy. Consequently, it is difficult to assess the impact of the interventions on women’s self-efficacy or empowerment, and to understand the association between empowerment and SRH outcomes. Not measuring other outcomes limits the evidence for pathways to improved health for women living with HIV and their partners. Additionally, studies used a wide range of measures for condom use that affected our ability to compare across interventions and precluded us from conducting meta-analysis. Condom use reported in these studies was affected by high rates of initial reported use, creating a ceiling for measuring intervention impact. Many measures were also self-reported, introducing the possibility of recall and social desirability bias. Finally, the included studies were of mixed quality, with many limited by small sample size and low follow-up rates. The evidence base is further limited in geographic and population scope. Many important populations of women living with HIV, such as transgender women, were not included in any studies. Most included studies were conducted in the USA or were adaptations of interventions originally implemented there. Nevertheless, some interventions were determined to be effective when adapted to multiple contexts and feasible across settings. Finally, we did not include unpublished (“grey”) literature or qualitative studies in our inclusion criteria; these studies may have provided additional insights into the effectiveness and outcomes of interventions.

Although this review focused on interventions with women, interventions with men that seek to address unequal gender and power relations are also essential to empower women in their SRH decisions. Recent evidence suggests that gender-transformative interventions to engaging men in HIV [42] and gender-based violence [43] hold promise; such programs seek to directly discuss and reconfigure gender roles in the direction of more gender equitable relationships [44]. Additionally, many gender inequalities exist at a structural level through cultural norms, laws, and institutions. Future research should also seek to implement structural-level interventions so that women may live in environments that better facilitate their control over their own sexual and reproductive health. Though structural-level interventions can be challenging both to implement and evaluate, they can have significant impact [45].

This is the first systematic review of interventions to improve self-efficacy and empowerment around safer sex and reproductive health decision-making for women living with HIV. The limitations of the existing evidence indicate a need for further research to determine the impact of empowerment and self-efficacy interventions. Future studies should include measurement of a wider range of sexual and reproductive health and rights outcomes, including both proximal empowerment and more distal health outcome measures. Studies should ensure the meaningful participation of the community of women living with HIV in study design. Interventions should also be explicit about how their content addresses unequal gender power relations. Such studies would allow for clear conclusions on how these types of interventions may improve the SRH of women living with HIV.

## Supporting information

S1 Appendix Full search strategy for PubMed. (DOCX)

S1 Checklist PRISMA checklist. (DOCX)
