# Supplementary material for: Interventions to address unequal gender and power relations and improve self-efficacy and empowerment for sexual and reproductive health decision-making for women living with HIV: A systematic review
Source: PLoS One. 2017 Aug 24;12(8):e0180699. doi: 10.1371/journal.pone.0180699 (PMC5570301; doi:10.1371/journal.pone.0180699)
Supplement: S1 Appendix — (DOCX) [file pone.0180699.s001.docx]

**S1 Appendix. Full search strategy for Pubmed.**

**Concept 1: HIV**

(“HIV positive” [tiab] OR “living with HIV” [tiab] OR “HIV infected” [tiab])

AND

**Concept 2: Women**

(Women’s Health[mesh] OR women[tiab] OR woman[tiab] OR female*[tiab] OR gender[tiab])

AND

**Concept 3: Study type**

(Intervention studies[Mesh] OR case control studies[mesh] OR case-control stud*[tiab] OR intervention*[tiab] OR evaluat*[tiab] OR evaluation studies as topic[MeSH] OR assess*[tiab] OR program evaluation[mesh] OR randomized controlled trial[pt] OR controlled clinical trial[pt] OR randomized[tiab]OR random*[tiab] OR clinical trials as topic[mesh:noexp] OR "non-randomized trial"[tiab] OR "pre post study"[tiab] OR "before after study"[tiab] OR "time series study"[tiab] OR "cross-sectional"[tiab] OR “double-blind procedure”[tiab] OR "single-blind procedure"[tiab] OR "retrospective cohort"[tiab] OR randomly[tiab] OR trial[ti] NOT (animals[mh] NOT humans[mh]))

AND

**Concept 4: SRH concepts**

(Self Efficacy[mesh] OR self concept[mesh] OR empower*[tiab] OR “self-efficacy”[tiab] OR “self esteem”[tiab] OR self perception*[tiab] OR “decision-making”[tiab] OR power[tiab] OR risk reduction behavior[mesh] OR “risk reduction”[tiab]).
