# Supplementary material for: Interventions to address unequal gender and power relations and improve self-efficacy and empowerment for sexual and reproductive health decision-making for women living with HIV: A systematic review
Source: PLoS One. 2017 Aug 24;12(8):e0180699. doi: 10.1371/journal.pone.0180699 (PMC5570301; doi:10.1371/journal.pone.0180699)
Supplement: S1 Checklist — (DOCX) [file pone.0180699.s002.docx]

| **Section/topic** | **#** | **Checklist item** | **Reported on page #** |
| --- | --- | --- | --- |
| **TITLE** | | |  |
| Title | 1 | Identify the report as a systematic review, meta-analysis, or both.  Title includes “a systematic review” | 1 |
| **ABSTRACT** | | |  |
| Structured summary | 2 | Provide a structured summary including, as applicable: background; objectives; data sources; study eligibility criteria, participants, and interventions; study appraisal and synthesis methods; results; limitations; conclusions and implications of key findings; systematic review registration number.  Abstract includes this information as applicable, and as space allowed. | 2-3 |
| **INTRODUCTION** | | |  |
| Rationale | 3 | Describe the rationale for the review in the context of what is already known.  Introduction includes the context of what is already known and notes, “However, the effectiveness of such interventions has not been systematically assessed.” | 4 |
| Objectives | 4 | Provide an explicit statement of questions being addressed with reference to participants, interventions, comparisons, outcomes, and study design (PICOS).  “We conducted a systematic review to examine the effectiveness of interventions that aim to address unequal gender power relations, empower women living with HIV, and increase their self-efficacy to make SRH decisions.” | 4 |
| **METHODS** | | |  |
| Protocol and registration | 5 | Indicate if a review protocol exists, if and where it can be accessed (e.g., Web address), and, if available, provide registration information including registration number.  “This systematic review followed PRISMA reporting guidelines; the review protocol is available upon request.” | 5 |
| Eligibility criteria | 6 | Specify study characteristics (e.g., PICOS, length of follow-up) and report characteristics (e.g., years considered, language, publication status) used as criteria for eligibility, giving rationale.  See section titled “Eligibility Criteria” | 5 |
| Information sources | 7 | Describe all information sources (e.g., databases with dates of coverage, contact with study authors to identify additional studies) in the search and date last searched.  See section titled “Data Sources” | 6 |
| Search | 8 | Present full electronic search strategy for at least one database, including any limits used, such that it could be repeated.  A full search strategy for PubMed is included in the Appendix; other information about the search is in the section entitled “Data Sources” | Appendix; 6 |
| Study selection | 9 | State the process for selecting studies (i.e., screening, eligibility, included in systematic review, and, if applicable, included in the meta-analysis).  “Initial screening of titles and abstracts was done by a single reviewer. Potentially relevant citations were then independently screened in duplicate and resolved through discussion. Full-text articles were reviewed for final eligibility decisions.” | 6 |
| Data collection process | 10 | Describe method of data extraction from reports (e.g., piloted forms, independently, in duplicate) and any processes for obtaining and confirming data from investigators.  “Data were independently extracted in duplicate using standardized forms. Differences in data extraction were resolved through discussion and consensus. | 6 |
| Data items | 11 | List and define all variables for which data were sought (e.g., PICOS, funding sources) and any assumptions and simplifications made.  “The following information was gathered from each included study: objectives, location, population characteristics, intervention description, study design, sample size, follow-up periods, loss-to-follow-up, analytic approach, outcome measures, comparison groups, effect sizes, confidence intervals, significance levels, conclusions, and limitations.” | 6 |
| Risk of bias in individual studies | 12 | Describe methods used for assessing risk of bias of individual studies (including specification of whether this was done at the study or outcome level), and how this information is to be used in any data synthesis.  “Rigor was assessed using the Evidence Project’s tool for evaluating multiple study designs in HIV behavioral intervention research [10], including assessment of comparison groups, random assignment and selection, follow-up rate, equivalency of comparison groups, and control for potential confounders.” | 6 |
| Summary measures | 13 | State the principal summary measures (e.g., risk ratio, difference in means). | N/A |
| Synthesis of results | 14 | Describe the methods of handling data and combining results of studies, if done, including measures of consistency (e.g., I^2^) for each meta-analysis.  “Data were descriptively analyzed by coding categories and SRH outcomes. We did not meta-analyze due to differences in intervention design and outcome measurement across studies. However, we grouped similar measures (e.g., condom use self-efficacy) across studies and summarized findings by outcome.” | 6 |

Page 1 of 2

| **Section/topic** | **#** | **Checklist item** | **Reported on page #** |
| --- | --- | --- | --- |
| Risk of bias across studies | 15 | Specify any assessment of risk of bias that may affect the cumulative evidence (e.g., publication bias, selective reporting within studies). | N/A |
| Additional analyses | 16 | Describe methods of additional analyses (e.g., sensitivity or subgroup analyses, meta-regression), if done, indicating which were pre-specified. | N/A |
| **RESULTS** | | |  |
| Study selection | 17 | Give numbers of studies screened, assessed for eligibility, and included in the review, with reasons for exclusions at each stage, ideally with a flow diagram.  “Database searches produced a total of 3,351 hits; 2,087 citations remained after removing duplicates (Fig 1). After initial screening, 151 citations were reviewed by two authors in duplicate, of which 73 were excluded for not meeting the inclusion criteria (e.g., qualitative studies, studies without relevant outcomes, or studies without findings for women living with HIV). Seventy-eight articles were pulled for full-text review, and 57 were excluded. Ultimately, 21 studies were included in the review covering 11 specific intervention approaches (Table 1).” | 7 |
| Study characteristics | 18 | For each study, present characteristics for which data were extracted (e.g., study size, PICOS, follow-up period) and provide the citations.  Table 1 | Table 1 (page 8) |
| Risk of bias within studies | 19 | Present data on risk of bias of each study and, if available, any outcome level assessment (see item 12).  Table 2 | Table 2 (page 13) |
| Results of individual studies | 20 | For all outcomes considered (benefits or harms), present, for each study: (a) simple summary data for each intervention group (b) effect estimates and confidence intervals, ideally with a forest plot.  Table 3 | Table 3 (page 15) |
| Synthesis of results | 21 | Present the main results of the review. If meta-analyses are done, include for each, confidence intervals and measures of consistency.  See “Study Descriptions” and “Study Outcomes” sections | 11-19 |
| Risk of bias across studies | 22 | Present results of any assessment of risk of bias across studies (see Item 15). | N/A |
| Additional analysis | 23 | Give results of additional analyses, if done (e.g., sensitivity or subgroup analyses, meta-regression [see Item 16]). | N/A |
| **DISCUSSION** | | |  |
| Summary of evidence | 24 | Summarize the main findings including the strength of evidence for each main outcome; consider their relevance to key groups (e.g., healthcare providers, users, and policy makers).  “All women living with HIV must be supported in their voluntary choices around sexual relationships and be given information and resources to engage in safe, enjoyable sexual experiences, or to not engage in sex based on their personal preference, with counselling and support tailored to their decision-making, desires and needs. Supporting women living with HIV in all their diversity to achieve their sexual and reproductive health and rights in all epidemic contexts requires overcoming major barriers to service uptake such as social exclusion and marginalization, criminalization, stigma, and gender inequality [37]. Addressing unequal gender and power relations and empowering women living with HIV may be one part of a comprehensive approach to achieve these goals.  This systematic review highlights the potential for increasing condom use and reducing incident STIs through empowerment interventions for women living with HIV. STIs continue to be an important public health issue that can facilitate sexual transmission of HIV and trigger some cancers. As stated in the WHO Global Health Sector Strategy on Sexually Transmitted Infections, 2016-2021, “the burden of morbidity and mortality worldwide resulting from sexually transmitted pathogens compromises quality of life, as well as sexual and reproductive health” [33]. Women living with HIV have high rates of STI co-infection, with a mean STI prevalence of 15.8% (standard deviation: 9.9) across studies in a recent global systematic review [34]. Although STI screening and treatment are a recommended part of the package of care for people living with HIV by the WHO [35, 36] and PEPFAR [37], a comprehensive, rights-based approach to addressing STIs and other SRH issues is needed to facilitate STI prevention as well as treatment for women living with HIV.  Findings from our review were more mixed, however, for other outcomes, including contraceptive use, self-efficacy, and psychosocial measures. While these interventions hold promise, further work is needed to determine which components of interventions make them successful, for which populations, and on which outcomes.” | 19-20 |
| Limitations | 25 | Discuss limitations at study and outcome level (e.g., risk of bias), and at review-level (e.g., incomplete retrieval of identified research, reporting bias).  “Conclusions from this review are limited by the nature of the evidence base. The range of outcomes measured by the included studies was narrow, with the majority measuring condom use. Only a few studies measured other SRH outcomes, or more proximal outcomes such as empowerment and self-efficacy. Consequently, it is difficult to assess the impact of the interventions on women’s self-efficacy or empowerment, and to understand the association between empowerment and SRH outcomes. Not measuring other outcomes limits the evidence for pathways to improved health for women living with HIV and their partners. Additionally, studies used a wide range of measures for condom use that affected our ability to compare across interventions and precluded us from conducting meta-analysis. Condom use reported in these studies was affected by high rates of initial reported use, creating a ceiling for measuring intervention impact. Finally, the included studies were of mixed quality, with many limited by small sample size and low follow-up rates. The evidence base is further limited in geographic and population scope. Many important populations of women living with HIV, such as transgender women, were not included in any studies. Most included studies were conducted in the USA or were adaptations of interventions originally implemented there. Nevertheless, some interventions were determined to be effective when adapted to multiple contexts and feasible across settings.” | 20-21 |
| Conclusions | 26 | Provide a general interpretation of the results in the context of other evidence, and implications for future research.  “This is the first systematic review of interventions to improve self-efficacy and empowerment around safer sex and reproductive health decision-making for women living with HIV. The limitations of the existing evidence indicate a need for further research to determine the impact of empowerment and self-efficacy interventions. Future studies should include measurement of a wider range of sexual and reproductive health and rights outcomes, including both proximal empowerment and more distal health outcome measures. Studies should ensure the meaningful participation of the community of women living with HIV in study design. Interventions should also be explicit about how their content addresses unequal gender power relations. Such studies would allow for clear conclusions on how these types of interventions may improve the SRH of women living with HIV.” | 21 |
| **FUNDING** | | |  |
| Funding | 27 | Describe sources of funding for the systematic review and other support (e.g., supply of data); role of funders for the systematic review.  Not included in manuscript, but presented separately, per PLoS Med requirements: “This study was funded by the World Health Organization, Department of Reproductive Health and Research. WHO commissioned this review to inform updated WHO guidelines on sexual and reproductive health and rights of women living with HIV. The funder contributed to the study design, data collection, analysis, interpretation, and writing of the review.” | N/A |

*From:*  Moher D, Liberati A, Tetzlaff J, Altman DG, The PRISMA Group (2009). Preferred Reporting Items for Systematic Reviews and Meta-Analyses: The PRISMA Statement. PLoS Med 6(7): e1000097. doi:10.1371/journal.pmed1000097

For more information, visit: **www.prisma-statement.org**.

Page 2 of 2
